# Parent–Child Relationships and Adolescents’ Non-Cognitive Skills: Role of Social Anxiety and Number of Friends

**DOI:** 10.3390/bs14100961

**Published:** 2024-10-17

**Authors:** Xiaoxue Kuang, Fen Ren, John Chi-Kin Lee, Hui Li

**Affiliations:** 1 School of Education, Dongguan University of Technology, Dongguan 523808, China; 2017057@dgut.edu.cn; 2 School of Education and Psychology, University of Jinan, Jinan 250022, China; sep_renf@ujn.edu.cn; 3 Department of Curriculum and Instruction, The Education University of Hong Kong, Hong Kong 999077, China; jcklee@eduhk.hk; 4 School of Education, Guangzhou University, Guangzhou 510006, China

**Keywords:** parent–child relationships, social anxiety, non-cognitive skills, number of positive and negative friends

## Abstract

This study aimed to examine the association between parent–child relationships and adolescents’ non-cognitive skills, while also investigating the mediating effect of social anxiety and number of friends. A survey was conducted with 773 students, ranging from grade 4 to 9, in five public schools of Guangdong Province of China (49.9% male), with a mean age of 12.20 years old. Latent mediation structural equation modeling was used to analyze the data. The findings revealed that (1) the father–child relationship and mother–child relationship both had a positive influence on grit, innovation, conscientiousness, and hope; (2) social anxiety had a negative effect on adolescent non-cognitive skills including innovation, conscientiousness, and hope; (3) the number of positive friends was found to be positively associated with the development of non-cognitive skills in adolescents, whereas the presence of negative friends correlated negatively with grit but positively with innovation; (4) social anxiety emerged as a significant mediating variable between parent–child relationships and adolescents’ non-cognitive skills, including innovation, conscientiousness, and hope; and (5) the mediating effect of the number of positive friends on the relationship between parent–child relationships and grit, innovation, conscientiousness, and hope was also found to be significant. Educational programs and family interventions should take these factors into account, providing a more holistic approach to supporting adolescent growth.

## 1. Introduction

The term non-cognitive skills (NCSs), also known as social and emotional skills [1], personality traits or character skills [2], encompasses a broad range of personal attributes, competencies, and characteristics. The definition and measurement of NCSs have posed challenges for researchers, as highlighted by Heckman and Kautz, who emphasized the lack of comprehensive indicators [3]. The concept of NCSs has been defined by numerous researchers, drawing upon personality traits derived from the “Big Five” framework encompassing openness to experience, conscientiousness, extraversion, agreeableness, and neuroticism [4]. Researchers believe the scope of NCSs has been broadened beyond the constraints of the Big Five framework, with their characterization now encompassing motivation, perseverance, self-control, social abilities or similar concepts [5,6,7]. Some scholars also argue that the concept of non-cognitive skills was introduced in contrast to cognitive skills, including grit, innovation, creative thinking, emotional control, social skills, and the quality of friends [8,9]. Due to the lack of standardized methods for measuring non-cognitive skills in academic circles, this study determined four variables for measuring non-cognitive skills based on reference to similar studies, namely grit, innovation, conscientiousness, and hope.

In the early 1970s, Bowles and Gintis posited that NCSs might be as crucial as cognitive abilities in explaining status attainment and academic performance [10,11,12]. Kautz et al. further argued that achievement tests could only explain a small fraction of the variance in later-life success, underscoring the significant value placed on NCSs in the labor market, schools, and society, despite being largely overlooked [2]. A growing body of empirical research has emphasized the significant influence of non-cognitive abilities on individuals’ educational outcomes, cognitive development, health, psychosocial outcomes, social well-being, and success in the labor market [12,13,14]. Studies have revealed that compared to adults, adolescents’ NCSs exhibit greater volatility and plasticity, making them more susceptible to external influences such as family dynamics, parent–child relationships, and peer interactions [15,16]. The impact of factors such as physical exercise, parental migration, marriage and parenting on children’s non-cognitive development has been examined in some studies [17,18,19,20], especially in early childhood, while for early adolescence, there are no consistent results. The study conducted by Grätz et al. revealed a significant impact of parenting style, parental activities, and extracurricular activities during early adolescence (10–14-year-olds) on the development of non-cognitive skills such as academic self-concept, motivation, self-esteem, self-efficacy, and locus of control [21]. Another study indicated a significant association between parenting style, parental investment, the self-esteem scale, locus of control, and emotion stability [20].

As pointed out by Del Bono et al. [22] previous studies often relied on parental assessments to measure children’s non-cognitive skills. However, these assessments can be influenced by the parents’ own skills, leading to measurement contamination. Few studies have thoroughly examined the mechanisms linking family relationships to non-cognitive abilities, particularly during the transition from childhood to early adolescence, while also considering the influence of peers. Therefore, the purpose of this study is to explore the relationships between positive NCSs, parent–child relationships, societal influences, and the number of friends. Of particular interest is elucidating the role of parent–child relationships in the development of these NCSs during this critical transition period. Given the broad scope of personal attributes encompassed by non-cognitive skills (NCSs) and the lack of a standardized measure for them, this study will only focus on four positive components of NCSs: grit, innovation, conscientiousness, and hope. These specific NCSs are selected because they are positively and morally valued traits indicative of good character [23]. Additionally, they explain variance in various work, educational, behavioral and health outcomes beyond the Big Five personality traits [24]. This study focuses on Chinese students as research subjects, aligning with the ongoing educational reforms in China that emphasize the development of adolescents’ non-cognitive skills. Traditionally, Chinese education has prioritized cognitive skills such as reading, writing, and arithmetic [25]. However, there is now a growing recognition among both schools and families regarding the significance of non-cognitive skills for adolescent development. Consequently, this study can also offer valuable insights for collaboration between Chinese schools and families in enhancing adolescents’ non-cognitive skills.

## 2. Literature Review

### 2.1. Parent–Child Relationship and Adolescents’ Non-Cognitive Skills

The family plays a pivotal role in society, providing children with essential life experiences, fostering interpersonal connections, transmitting social norms, and shaping behaviors and personalities [26]. Drawing upon Bronfenbrenner and Morris’s bioecological model of human development, the family operates as a microsystem comprising activities and interactions that profoundly impact children’s development. Within this framework, the relationship between parents emerges as a critical factor influencing child development [27]. According to McMaster’s model of family functioning [28], family members are interconnected, and each individual’s actions and experiences are intertwined with and influenced by other family members. Family dynamics are shaped not only by individual characteristics but also by patterns of communication and interaction within the family system. Family closeness, a significant aspect of family functioning, plays a vital role in shaping children’s development [29]. Parent–child relationships, as a key manifestation of family closeness, not only directly impact children’s academic performance but also contribute to their emotional, affective, and personality development [30,31,32].

The existing literature consistently demonstrates the positive impact of the parent–child relationship on various aspects of individual development. Specifically, studies have shown that a strong parent–child relationship is associated with higher levels of conscientiousness [33], increased social creativity [34], enhanced adolescent self-worth [35], greater resilience [36,37], and lower levels of neuroticism [33]. Conversely, poor parent–child relationships characterized by parental monitoring, psychological control, and rejection/hostility have been linked to delinquent behaviors in children [38]. Furthermore, Suzuki et al. [6] found that adolescents aged 14–15 with positive parent–child relationships reported higher levels of NCSs (grit and self-control). Gender also plays a role in the association between parent–child relationships and non-cognitive skills. For male adolescents, a secure attachment to their father was linked to healthier personality development, greater ego resilience, and lower levels of perceived hostility by friends, while for female adolescents, closeness in the mother–child relationship was negatively related to ego resilience [32]. Moreover, parent–child attachment has been shown to influence children’s social interactions, with strong attachments to both parents predicting positive peer attachments, particularly with the same-sex parent [39,40]. Therefore, it is crucial to consider the distinct role of parent gender in this study.

Another important aspect to consider regarding parent–child relationships is that they undergo changes during the transition to adolescence, particularly from primary school to secondary school. During the transition period, the amount of time parents and youths spend together decreases. Especially in the context of Chinese culture, middle school students are required to attend evening study sessions and may even stay at school in dormitories, resulting in less time at home and significantly less time with their parents compared to primary school students. Meanwhile, adolescents develop an increased desire for autonomy from their parents, which may lead to a reduction in closeness between parents and children [41,42]. Studies have indeed shown declines in feelings of closeness to parents from early to mid-adolescence. Given these dynamics, it is crucial to explore the relationship between parent–child relationships and NCSs while considering the possible effects of parents’ gender and students’ age, particularly from early to mid-adolescence. The objective of this paper is to establish connections between parent–child relationships and children’s and adolescents’ NCSs, focusing specifically on positive aspects such as grit, conscientiousness, innovation, and hope.

### 2.2. Mediating Effect of Number of Friends

The quantity and quality of friendships have been extensively studied as crucial aspects of peer relationships contributing to positive development during adolescence [43]. Barry and Wentzel [44] found that a friend’s behavior, including affective quality and interaction frequency, was associated with an individual’s pursuit of prosocial goals, which, in turn, influenced their prosocial behavior. Wagner demonstrated a positive relationship between friendship quality and various character strengths among early adolescents [25]. Additionally, Wagner found that curiosity, love of learning, and spirituality were negatively correlated with the number of mutual friends, indicating that having fewer mutual friends may allow for a deeper exploration of personal interests [25]. The number of mutual friends—those who have both nominated each other—has been shown to uniquely align with peer-rated prosocial skills [45].

Previous studies have primarily utilized nominated methods to quantify the number of mutual friends, which, however, did not differentiate whether the friend was beneficial or not. Friends can be categorized as either good or poor, with good friends positively impacting personal growth and poor friends potentially having the opposite effect. However, limited research has considered the distinction between good and poor friends. Moreover, studies have highlighted that parent–child connectedness is correlated with children’s socioemotional orientations, the number of mutual friendships, and peer friendships [46]. Therefore, this study assumes that the number of positive and negative friendships may have different effects on non-cognitive skills and could mediate the association between parent–child relationships and non-cognitive skills.

### 2.3. Mediating Effect of Social Anxiety

Social anxiety is an emotional state of anxiety characterized by heightened feelings of anxiety triggered by the anticipation or experience of interpersonal evaluation in either real or imagined social situations. This condition significantly impacts an individual’s mental well-being and social relations [47]. Numerous studies have investigated the relationship between the five-factor personality traits and social anxiety, consistently identifying high neuroticism and low extraversion as prominent predictors of social anxiety across cultures [48,49,50].

Furthermore, some studies have highlighted the relationship between social anxiety and several personality traits at the phenotypic, genetic, and environmental levels [51]. However, the effects of anxiety disorders on personality have received less attention. Karsten et al. [52] examined changes in all five personality traits associated with the occurrence or recovery from anxiety disorders, finding that neuroticism trait scores increased with the occurrence of anxiety disorders and decreased with recovery from depressive and anxiety disorders, based on data from an 8-year longitudinal cohort study with an adult sample. Prince et al. [53] suggested that anxiety disorders have the potential to confound personality assessment. They found that high neuroticism in young adulthood is either a true risk factor or a marker of risk for first-onset anxiety and depressive disorders, as is low extraversion for later agoraphobia.

Kaplan et al. [54] discovered that social anxiety is negatively correlated with trust (a facet of agreeableness) and self-efficacy (a facet of conscientiousness). Anxiety disorders have been found to be negatively associated with trust in agreeableness and actions in openness, and positively associated with fantasy in openness [55]. Additionally, Costache et al. observed that individuals with Social Anxiety Disorder (SAD) exhibited higher scores for neuroticism and significantly lower scores for extraversion, openness, and conscientiousness compared to healthy counterparts [49]. While prior studies have primarily focused on adult samples (aged 18 and above) or college students, there has been limited exploration into the association between social anxiety and NCSs among adolescents. However, it is worth noting that social anxiety could potentially impact an individual’s NCSs during adolescence as well. Moreover, research has indicated that poor parent–child relationships characterized by rejection, over-control, and low parental warmth are associated with higher levels of social anxiety. Conversely, good parent–child relationships, characterized by social support, acceptance, and validation have been linked to lower levels of youth social anxiety [56,57].

Based on prior reviews, this study posits the hypothesis that social anxiety may impact the NCSs of adolescents and potentially act as a mediating factor in the relationship between parent–child relationships and NCSs.

### 2.4. Research Hypotheses

Previous research has underscored that the transition from childhood to adolescence instigates changes in personality traits, marking a pivotal period of psychological development for adolescents. Understanding the intricate relationships between parent–child relationships and adolescent non-cognitive skills is imperative, yet our current comprehension remains limited. Particularly, there is a dearth of empirical evidence concerning the complicated relationships among these variables within the context of Chinese education. According to Ainsworth and Bowlby’s Attachment Theory (1991), the relationship between a child and their primary caregiver forms the foundation for their social, emotional, and cognitive development [58]. This bond with parents fosters the child’s NCSs, which may facilitate friendship formation and reduce social anxiety. Additionally, from the perspective of Bronfenbrenner’s Ecological Systems Theory (1979), the parent–child relationship, social anxiety, and friendships are conceptualized as interacting components within the child’s broader social ecology, collectively contributing to the development of NCSs [59].

Therefore, the purpose of this study is to investigate the relationships between parent–child relationships, non-cognitive skills, societal anxiety, and the number of friends, with a specific emphasis on elucidating the role of parent–child relationships in shaping non-cognitive skills during adolescence. To address these critical inquiries, we employ a latent mediation structural model to elucidate the predictive associations between parent–child relationships and the development of positive traits among Chinese primary and middle school students. The findings offer both theoretical insights and practical implications for future research endeavors.

**Figure 1:**
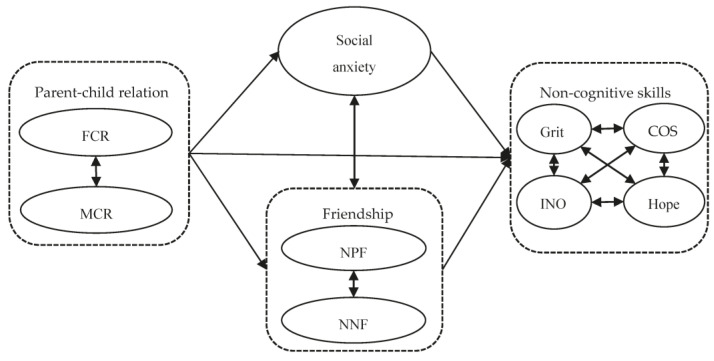
Conceptual model diagram. Note. FCR: Father–child relationship; MCR: Mother–child relationship; INO: Innovation; COS: Conscientiousness; NPF: number of positive friend; NNF: number of negative friend.

As depicted in Figure 1, the study presents a conceptual model and posits the following research hypotheses:

**Hypothesis 1.**  *There exists a significant positive correlation between parent–child relationships and non-cognitive skills, and a notable negative correlation between social anxiety and non-cognitive skills among Chinese adolescents.*

**Hypothesis 2.**  *A substantial positive correlation is observed between the number of positive friends and non-cognitive skills, alongside a significant negative correlation between the number of negative friends and non-cognitive skills among Chinese adolescents.*

**Hypothesis 3.**  *Social anxiety and the number of friends mediate the relationship between parent–child relationships and non-cognitive skills.*

## 3. Materials and Methods

### 3.1. Subjects

The data were collected from a sample of 773 students enrolled in five public schools spanning from grade 4 to grade 9, with a mean age of 12.20 (SD = 1.61), situated in Guangdong Province, China, employing a convenient sampling technique. The final sample comprised 386 male students (49.9%) and 386 female students (49.9%), with one individual missing gender information (0.1%). Among the participants, 272 students attended public primary schools (35.2%), while 501 students were enrolled in public secondary schools (60.8%).

### 3.2. Measures

#### 3.2.1. Parent–Child Relationships

The study utilized the Parent–Child Relationship Scale, originally developed by Buchanan et al. [60] and later translated and revised by Zhang et al. [61]. This scale, consisting of nine items, was employed to assess the individuals’ parent–child relationships. However, in this study, only seven items were utilized due to results from exploratory factor analysis (EFA) indicating that the factor loading and commonalities of two items were below 0.5.

The father–child relationship and mother–child relationship were measured separately, each with seven items featuring identical content, albeit referred to differently. Each item was rated on a five-point Likert scale ranging from 1 (“never”) to 5 (“always”), with higher scores indicating a more positive parent–child relationship. The Cronbach’s alphas for the father–child and mother–child scales were 0.89 and 0.85, respectively. Model fit indices demonstrated satisfactory results for both the father–child scale (CFI = 0.96, TLI = 0.95, RMSEA = 0.09, SRMR = 0.03) and the mother–child scale (CFI = 0.95, TLI = 0.93, RMSEA = 0.12, SRMR = 0.03).

#### 3.2.2. Non-Cognitive Skills

Grit, as measured by Zheng [62], was assessed using three items, such as “Even if it’s a subject I don’t like, I will do my best”. The Cronbach’s alpha for the scale was 0.76.

Innovation, also measured by Zheng [62], was evaluated through three items, including “I am very curious about new things”. The Cronbach’s alpha for this scale was 0.69.

Conscientiousness, as operationalized by Zheng [62], comprised six items, such as “I only play after completing school assignments”. In this survey, the scale exhibited a reliability of 0.83. Participants responded to these three scales using a four-point Likert rating ranging from 1 (“not like me”) to 4 (“very much like me”), with higher scores indicating higher levels of these traits.

The Children’s Hope Scale [63,64] consisted of six items—for example, “I think I’m doing well”. In this survey, the scale demonstrated a reliability of 0.89. Each item was rated on a six-point Likert scale (1 = “never” 6 = “always”), with higher scores reflecting a higher level of hope.

The model fit for the four-factor structure of NCSs was satisfactory in this study (CFI = 0.94, TLI = 0.93, RMSEA = 0.06, SRMR = 0.04).

#### 3.2.3. Social Anxiety Scale of Children (SASC)

The Social Anxiety scale, originally developed by La Greca [65] and translated into Chinese by Li et al. [66], comprises 10 items and is widely used to assess social anxiety in children aged 7 to 16 (e.g., “I am afraid to do things in front of others that I haven’t done before). Participants rate each item on a three-point scale (0 indicates “never”, 1 indicates “sometimes”, and 2 indicates “always”), with higher scores indicative of higher levels of social anxiety. The scale demonstrated strong internal consistency, with a Cronbach’s alpha of 0.87. The model fit for the one-factor structure of social anxiety was deemed satisfactory in this study (CFI = 0.95, TLI = 0.94, RMSEA = 0.11, WRMR = 1.42).

#### 3.2.4. Number of Friends

Positive friends were measured using three items (e.g., “How many of your best friends have excellent academic performance?”). Similarly, negative friends were evaluated through three items (e.g., “How many of your best friends violate school discipline?“) [62]. Participants respond on a scale with three-point rating (1 indicates “0”, 2 indicates “1–2”, and 3 indicates “≥3, equal to or more than three”). The Cronbach’s alpha for the positive friends and negative friends scales were 0.78 and 0.77, respectively. The model fit for the two-factor structure of the friends scale was good (CFI = 0.99, TLI = 0.98, RMSEA = 0.05, SRMR = 0.03).

### 3.3. Procedure

Participants provided informed consent in person, and this study strictly adhered to all ethical and consent procedures. The research content posed no harm to the participants. With the permission of school authorities, the study selected two primary and three secondary schools in the county and sampled 773 adolescents by including whole classes.

### 3.4. Statistical Analysis

Basic analysis of the data involved calculating frequencies, percentages, means, and standard deviations (SDs). The relationship between variables was analyzed using the Pearson product-moment correlation in IBM SPSS version 27. Additionally, latent mediate structural equations were conducted using Mplus 8.0 for further analyses.

## 4. Results

### 4.1. Descriptive Statistics

Table 1 displays descriptive statistics, including correlation coefficients, the mean scores, standard deviations and other indicators, for parent–child relationships, NCSs, social anxiety and number of friends. The mean scores for parent–child relationships with father and mother were 3.64 and 3.75, respectively. The data indicate a moderate level of closeness in parent–child relationships, with slightly higher levels of closeness reported with mothers compared to fathers. Students reported slightly higher scores for grit (*m* = 3.27) compared to innovation (*m* = 3.11) and conscientiousness (*m* = 3.10) on a scale with a maximum score of four. The level of hope was also moderate (*m* = 4.20) on a scale with a maximum score of four. Social anxiety among the entire sample was low, with a mean of 0.76. Regarding the number of friends, adolescents reported having more positive friends (*m* = 2.66) than negative friends (*m* = 1.54) on average. Meanwhile, the parent–child relationship of adolescents shows a decline between primary school and secondary school, with the average score for father–child relationship (FCR) dropping from 3.85 to 3.52 and for mother–child relationship (MCR) dropping from 3.90 to 3.67. The grit (*m*_p_ = 3.34; *m*_s_ = 3.23), innovation (*m*_p_ = 3.23; *m*_s_ = 3.04), conscientiousness (*m*_p_ = 3.21; *m*_s_ = 3.04) and hope (*m*_p_ = 4.26; *m*_s_ = 4.17) scores also declined when comparing students from primary than those from secondary school. It is noteworthy that there is a slight upward trend in the number of positive friends across grades, with the average score increasing from 2.60 in primary school to 2.70 in secondary school. Additionally, according to independent sample *t*-tests, there were significant differences in the MCR (*t* = 4.82, *p* < 0.001), FCR (*t* = 4.19, *p* < 0.001), grit (*t* = 2.50, *p* < 0.05), innovation (*t* = 4.30, *p* < 0.001), conscientiousness (*t* = 4.15, *p* < 0.001) and number of positive friends (*t* = −2.99, *p* < 0.01) between primary school and secondary school.

Male participants reported higher scores for FCR (*m*_m_ = 3.73; *m*_f_ = 3.55; *t* = 2.58, *p* < 0.05), innovation (*m*_m_ = 3.18; *m*_f_ = 3.03; *t* = 3.46, *p* < 0.001), hope (*m*_m_ = 4.30; *m*_f_ = 4.11; *t* = 2.31, *p* < 0.05) and number of negative friends (*m*_m_ = 1.74; *m*_f_ = 1.35; *t* = 10.90, *p* < 0.001). Males reported lower social anxiety than females (*m*_m_ = 0.66; *m*_f_ = 0.86; *t* = −6.14, *p* < 0.001). No significant gender differences were found in the other variables.

Table 1 also presents the correlation coefficients between parent–child relationships, NCSs, social anxiety and number of friends. Following Cohen’s guidelines, correlation coefficients of 0.1, 0.3, and 0.5 are considered to represent small, medium, and large effect sizes, respectively [67]. The data reveal significant positive correlations (*p* < 0.01) between the MCR, NCSs, and the number of positive friends. Additionally, significant negative correlations (*p* < 0.01) are observed between the MCR and social anxiety. However, all effect sizes were small (all *r*s below 0.3). Similar patterns are observed for the FCR, with effect sizes ranging from small to medium and absolute values of correlation coefficients ranging from 0.11 to 0.30. There is a significant weak negative correlation (*r* = −0.15, *p* < 0.01) between the MCR and number of negative friends.

### 4.2. Direct Effect

Harman’s single factor test was used to test the common method the variance [68]. The first common factor explained 11.14% of the total variance, which is below the critical value of 40%. Additionally, the one-factor model, which included all the variables, was tested using CFA. The model fit was very poor (CFI = 0.446, TLI = 0.424, RMSEA = 0.099), indicating no significant issue of common method bias in this study.

To test the direct effect of the parent–child relationships on NCSs, this study initially controlled for two variables: gender and grade level. The results showed that the model fit index was acceptable (χ^2^/*df* = 4.46, CFI = 0.908, TLI = 0.901, and RMSEA = 0.041, 90%CI [0.040–0.042]). The FCR and MCR significantly and positively predicted grit (*β*_1_ = 0.099, *p* < 0.01; *β*_2_ = 0.101 *p* < 0.01), innovation (*β*_1_ = 0.142, *p* < 0.001; *β*_2_ = 0.070, *p* < 0.05), conscientiousness (*β*_1_ = 0.182, *p* < 0.001; *β*_2_ = 0.128, *p* < 0.001) and hope (*β*_1_ = 0.158, *p* < 0.001; *β*_2_ = 0.183, *p* < 0.001). Social anxiety significantly and negatively predicts innovation (*β* = −0.134, *p* < 0.001), conscientiousness (*β* = −0.053, *p* < 0.05) and hope (*β* = −0.204, *p* < 0.001), as can be seen in model 1 of Table 2. The association between social anxiety and grit is not significant. Therefore, Hypothesis H1 was tested.

The number of positive friends significantly positively predicts grit (*β*_1_= 0.287, *p* < 0.001, innovation (β = 0.224, *p* < 0.001), conscientiousness *(β* = 0.275, *p* < 0.001) and hope (*β* = 0.222, *p* < 0.001). The number of negative friends significantly and negatively predicts grit (*β* = −0.137, *p* < 0.001) and conscientiousness (*β* = −0.155, *p* < 0.001). The associations between the number of negative friends and innovation and hope were not significant (see model 1 in Table 2). Therefore, Hypothesis H2 was tested.

### 4.3. Mediation Model Testing

To further explore the mechanism of the role of parent–child attachment in adolescent’s NCSs, social anxiety and the number of friends were included as mediating variables in the model for testing. The results indicated that the model fitted well (χ^2^/*df* = 3.685, CFI = 0.929, TLI = 0.923, and RMSEA = 0.036, 90%CI: 0.035–0.037). The mediating effect analysis was conducted using a deviation-corrected percentile bootstrap test (bootstrap = 5000), which calculated the estimates with 95% confidence interval in this study. If the confidence interval includes 0, the coefficient is deemed insignificant.

The FCR and MCR significantly and positively predicted grit (*β*_1_ = 0.101; *p* < 0.01; *β*_2_ = 0.102, *p* < 0.01), conscientiousness (*β*_1_ = 0.184, *p* < 0.001; *β*_2_ = 0.130, *p* < 0.001) and hope (*β*_1_ = 0.160, *p* < 0.001; *β*_2_ = 0.186, *p* < 0.001). Only the FCR positively predicted innovation (*β*_1_ = 0.144, *p* < 0.001). Social anxiety significantly and negatively predicted innovation (*β* = −0.137, *p* < 0.001) and hope (*β* = −0.209, *p* < 0.001). The number of positive friends significantly and positively predicted grit (*β* = 0.289, *p* < 0.001), innovation (*β*_1_ = 0.226, *p* < 0.001), conscientiousness (*β*_1_ = 0.277, *p* < 0.001) and hope (*β*_1_ = 0.224, *p* < 0.001). The number of negative friends significantly and negatively predicted grit (*β* = −0.150, *p* < 0.001) and conscientiousness (*β* = −0.169, *p* < 0.001) (see model 2 in Table 2). The correlation between social anxiety and NPF was 0.084 (*p* < 0.01), while the correlation between social anxiety and NNF was −0.083 (*p* < 0.01).

The results indicated that social anxiety significantly mediates the relationship between parent–child relationships and NCSs, specifically innovation and hope. For the relationship between the FCR and innovation, the 95% confidence interval (CI) for the mediating effect of social anxiety was [0.013, 0.043], with a mediating effect value of 0.026. For the relationship between father–child relationships and hope, the CI was [0.024, 0.060], with a mediating effect value of 0.040. Both CIs do not include 0, indicating significant mediating effects. Similarly, for the relationship between the MCR and innovation, the CI for the mediating effect of social anxiety was [0.002, 0.025], with a mediating effect value of 0.012. For the relationship between the MCR and hope, the CI was [0.004, 0.035], with a mediating effect value of 0.019. Again, both CIs do not include 0, confirming significant mediating effects (see model 2 in Table 2).

The results also demonstrated indirect effect of the FCR on grit, innovation, conscientiousness and hope through the number of positive friends (*β*_1_ = 0.046, 95%CI [0.022–0.070]; *β*_2_ = 0.036, 95%CI [0.017–0.056]; *β*_3_ = 0.044, 95%CI [0.022–0.067]; *β*_4_ = 0.035, 95%CI [0.017–0.056]) (See model 2 in Table 2). Similarly, the indirect effect of the MCR on grit, innovation, conscientiousness and hope through the number of positive friends was also significant (*β*_1_ = 0.024, 95%CI [0.001–0.048]; *β*_2_ = 0.019, 95%CI [0.001–0.038]; *β*_3_ = 0.023, 95%CI [0.001–0.046]; *β*_4_ = 0.019, 95%CI [0.001–0.038]). Additionally, significant indirect effects of the FCR and MCR on grit (*β*_1_ = 0.016, 95%CI [0.005–0.029]; *β*_2_ = 0.022, 95%CI [0.009–0.038]) and conscientiousness (*β*_1_ = 0.018, 95%CI [0.005–0.032]; *β*_2_ = 0.025, 95%CI [0.012–0.041]) through the number of negative friends were found. These findings indicate that parent–child relationships not only directly predict NCSs but also affect NCSs through the number of positive and negative friends. Therefore, Hypothesis H3 is verified.

## 5. Discussion

The primary objective of this study was to investigate the relationships between parent–child relationships, adolescent non-cognitive skills (NCSs), and the mediating roles of social anxiety and the number of friends. Overall, our findings suggest that the integrated mediation model was well-supported. Specifically, the direct effects of parent–child relationships on adolescent NCSs were statistically significant. Additionally, the indirect effects of both social anxiety and the number of friends on adolescent NCSs were also statistically significant after controlling for gender and grade. Previous research on adolescents has mainly focused on the association of NCSs with academic achievement. Our study suggests that interventions aimed at improving parent–child relationships may not only promote NCSs but would also increase the number of positive friends and reduce social anxiety in children. Therefore, enhancing parent–child relationships holds the potential to guide children toward positive paths of personal growth.

Aligned with Miller’s family functioning model [28] and Bronfenbrenner and Morris’s bioecological model [27], parent–child relationships serve as a fundamental cornerstone for individual development. Acting as a protective factor, parental support significantly contributes to the cultivation of an individual’s NCSs [68], serving as a robust support system to foster adolescent NCSs. Despite the potential weakening of parental influence during adolescence, as teenagers increasingly seek autonomy and spend less time with parents, maintaining closeness with parents remains pivotal as an external source of adolescent NCSs.

This study identified statistical differences in parent–child relationships and non-cognitive skills (NCSs), including grit, innovation, and conscientiousness, between primary and secondary school students. The parent–child relationship and NCSs exhibited a declining trend from primary to secondary school, aligning with previous research that observed similar declines in parent–child relationship quality [69] and NCSs such as grit [70] and conscientiousness [33,70] among secondary school students. The transition from childhood to adolescence, particularly from primary to middle school, is characterized by heightened self-consciousness and a quest for autonomy. Adolescents begin to assert their independence, leading to reduced interaction with parents as they spend more time at school, often engaging in evening self-study sessions or even residing on campus. This decreased interaction may contribute to a decline in the closeness of parent–child relationships [33]. Additionally, the decline in conscientiousness may stem from the significant academic pressure prevalent in the Chinese education system, particularly as students in secondary school face the challenges of the Junior High School Entrance Exam.

Contrary to expectations, no gender differences were found for grit, despite previous studies often reporting higher grit scores among girls than boys. However, boys scored higher for the father–child relationship, consistent with the observation by Starrels that fathers tend to be more involved with their sons [71]. Additionally, boys scored higher on innovation, hope, and the number of negative friends and exhibited lower social anxiety compared to females. While empirical studies on gender differences in innovation or creative ability have yielded inconsistent findings, our study provides further support for the notion that men outperform women in innovation, as reported in some previous studies [72,73]. Furthermore, Heaven and Ciarrochi [74] found that girls’ hope decreased during secondary school and remained consistently lower than boys’, possibly due to the influence of gender stereotypes that portray girls as less effective than boys. Adolescent girls, in particular, are subjected to a myriad of often conflicting messages about their roles and positions in society, stemming from family, media, and traditional Chinese culture. This finding aligns with previous research indicating that females experience significantly more social anxieties compared to males, possibly because girls place greater importance on social status and peer relationships, making them more sensitive to interpersonal stressors [75,76].

Additionally, in line with our expectations and consistent with previous research [77,78], this study found that both mother–child and father–child relationships were significantly and positively associated with the non-cognitive skills of adolescents, even after controlling for gender and grade effects. While there are slightly differing effects of father–child and mother–child relationships on the four variables, the father–child relationship exerted a stronger influence on children’s innovation and conscientiousness, whereas the mother–child relationship had a greater impact on hope. Previous research suggests that, compared to mothers, fathers are more likely to engage in activities that promote exploration, autonomy, and risk-taking—factors that are key contributors to innovation [79]. Fathers also tend to model goal-directed behavior, which may explain the effect of father–child relationship on conscientiousness [80]. On the other hand, mothers typically play a more significant role in fostering emotional support and perseverance, which are critical components in developing traits like hope and grit [81,82].

Social anxiety was found to significantly and negatively predict innovation, conscientiousness, and hope. High levels of social anxiety can impair cognitive flexibility and creative thinking, which are essential for innovation. Individuals with social anxiety tend to avoid social interactions and risk-taking behaviors, both of which are crucial for generating new ideas and engaging in creative problem-solving [83]. Additionally, social anxiety may reduce conscientiousness by leading to difficulties in task management, goal setting, and sustained focus on responsibilities, as anxiety often interferes with attention and self-discipline [84]. Furthermore, social anxiety can undermine hope by reducing goal-directed thinking and positive expectations for the future, as high levels of anxiety tend to foster pessimism and self-doubt. This is in line with Snyder’s hope theory, which highlights the importance of goal-directed thinking in fostering hope [82].

The number of positive friends was found to correlate with higher levels of grit, innovation, conscientiousness, and hope. Positive peer relationships offer essential emotional and academic support that fosters these traits. For instance, research has demonstrated that peer support significantly contributes to the development of grit, defined as perseverance and a passion for long-term goals [85]. Friends who demonstrate diligence and academic aspirations can also encourage similar behaviors in others, thereby enhancing conscientiousness. This is supported by Wentzel et al. [86], who emphasized the role of peers in promoting responsibility and goal-oriented behaviors. Additionally, peer collaboration and support have been shown to stimulate creativity, idea generation, and problem-solving, fostering innovation [87]. Positive friendships also provide emotional reinforcement, which can boost hope by promoting optimism and goal-directed thinking, as explained by Snyder et al. [82] in their hope theory. Interestingly, this study also found that the number of negative friends positively correlated with innovation. In our study, a ‘positive’ friend refers to someone who exerts a constructive influence on academic performance and behavior, such as through excellent academic achievements, diligent effort, and aspirations for higher education. Conversely, a ‘negative’ friend displays the opposite tendencies, including breaking rules, receiving reprimands, or getting into fights. These behaviors, particularly those linked to risk-taking and defying social norms, may contribute to innovation. Research has shown that innovation is not only fostered by constructive behaviors but can also emerge from non-conformity, risk-taking, and challenging the status quo [88]. Thus, while negative behaviors may hinder academic performance, they could still promote creative thinking and unconventional approaches, which are key elements of innovation.

The results indicated that social anxiety significantly mediates the relationship between parent–child relationships and NCSs, particularly innovation and hope. Parent–child relationships are known to shape children’s social and emotional development, and poor parent–child interactions can contribute to heightened levels of social anxiety [89]. Social anxiety, in turn, can impair cognitive flexibility, creativity, and innovation by restricting the individual’s willingness to take risks and engage in exploratory behaviors, which are critical for innovation [83]. Similarly, social anxiety can affect hope by reducing an individual’s ability to set and pursue goals, a core aspect of Snyder’s [84] hope theory. High levels of social anxiety may limit positive expectations for the future, thus undermining hope. Therefore, social anxiety acts as a mediator, affecting how parent–child relationships influence key aspects of NCSs like innovation and hope.

The results also demonstrated a significant indirect effect of the father–child relationship on grit, innovation, conscientiousness, and hope through the number of positive friends. Similarly, the mother–child relationship had a significant indirect effect on these traits through the same mechanism. Research suggests that positive parent–child relationships play a critical role in shaping children’s social competence, which in turn helps them form and maintain supportive peer relationships. Positive friends can reinforce traits like perseverance and goal-setting, contributing to higher levels of grit [82], and their influence on academic diligence and responsibility can promote conscientiousness [88]. In addition, positive peer environments encourage creativity and innovation through collaborative problem-solving and idea-sharing [87]. Moreover, these friendships provide emotional support that can enhance hope by fostering optimism and goal-directed thinking [82]. Thus, the quality of the parent–child relationship indirectly influences grit, innovation, conscientiousness, and hope through its impact on the child’s social relationships.

Our study provides valuable insights for families, educators, and psychological professionals. Furthermore, this study extends previous findings by demonstrating the combined contributions of parent–child relationships, social anxiety, and number of friends on four components (grit, conscientiousness, innovation, and hope) of adolescent NCSs outcomes within the same model. This integrated approach enhances our understanding of the complex interplay between these variables and their impact on adolescent development. Moreover, upon comparing the results across all the models, it becomes evident that the number of positive friends has the most significant impact on adolescent NCSs, even surpassing the effects of gender and grades. These findings align with the extensive body of literature highlighting the pervasive and crucial influence of peer relationships on various aspects of adolescent development.

It is also noteworthy that social anxiety and the number of friends partially mediated the associations between parental relationships and NCSs. This suggests that not only do parent–child relationships directly impact adolescent NCSs, but they also exert their influence indirectly through their effects on social anxiety and peer relationships. According to bioecological systems theory [27], individuals are deeply influenced by the environment in which they are embedded, as they interact with various elements of their surroundings. For adolescents, their family members within the household, teachers, and peers at school constitute the most significant components of their developmental environment, all of which may interact to shape their growth and development [79]. Consequently, parent–child relationships and friendships, being intimate relationships crucial to adolescent development, hold substantial importance. As adolescents transition from primary to secondary school, their emotional bonds with peers tend to strengthen. Positive peer relationships are known to have a constructive impact on children’s development [90]. Therefore, the number of positive friendships at school could potentially play a significant mediating role in parent–child relationships and NCSs.

## 6. Implications

The presented findings have valuable insights for educational practice. Through empirical analysis, this study elucidated the contributions of both mother–child and father–child relationships in fostering adolescent’s NCSs development within the Chinese educational context. In traditional culture of China, it is usually mothers who take care of the children and accompany them, and fathers often play the role of earning money, which is a marginalized or even absent position in family education [91]. With the progress of time and the development of society, more and more fathers are becoming aware of the importance of family education and actively involving themselves in their children’s education. The study demonstrated that the effect of a close father–child relationship on conscientiousness, innovation and grit was even larger when compared to the mother–child relationship. Therefore, it is crucial to encourage fathers to be actively involved in intimate interactions and communication with their children, alongside mothers. Sharing caregiving responsibilities between both parents fosters a sense of partnership and shared responsibility in parenting, which helps to create a more equitable and harmonious atmosphere within the household. This is not only in line with the traditional Chinese cultural values of honoring the family and fostering prosperity in all endeavors but also aligns with the requirements of the law on the promotion of Family Education promulgated by the Ministry of Education of the People’s Republic of China [92], which emphasizes the paramount importance of direct parenting and participation by both parents, leveraging the roles of both mother and father.

At the same time, a close parent–child relationship is essential to help children reduce social anxiety and promote the formation of more positive friendships at school. That is, when children have a close bond with their parents, they will feel emotionally secure and supported, which could help them reduce anxiety and provide a buffer against the stressors of social interactions at school. If parents demonstrate warmth, empathy, and respect in their interactions with their children, they might model these behaviors and emulate them their relationships with peers at school, which could help them form good friendships at school. A child’s lower social anxiety and increasing number of positive friends could enhance their positive non-congestive skills such as grit or conscientiousness. Certainly, further research should include comparative studies across diverse regions (western or eastern regions) and educational backgrounds (city or rural schools) of China, exploring the mechanisms through which father–child and mother–child relationships affect adolescents’ NCSs. These findings further enhance our understanding of the role parent–child relationships play in shaping the development of NCSs among primary and secondary school students. Moreover, the study offers valuable insights within the framework of the Chinese educational context.

## 7. Limitations

Firstly, it is important to acknowledge that the information on parent–child relationships, NCSs, social anxiety and number of friends used in this study was obtained from adolescents’ self-report questionnaires. While self-report measures can provide insights into adolescents’ actual closeness with parents and friends, they are also inherently prone to certain biases [33]. Future research, if feasible, could consider applying multiple methods and incorporating multiple sources of information—for example, in-depth interviews or direct observations with students, their peers, teachers and their parents—to assess parent–child and peer relationships, thereby obtaining a more comprehensive and accurate understanding of this topic. Secondly, this study employed a cross-sectional design, which only allows for the assessment of correlations among variables. Future research could conduct longitudinal studies, which enable the exploration of dynamic patterns of these variables over a long period. Moreover, longitudinal studies can help in measuring and controlling for unsystematic (and systematic) fluctuations in NCSs changes [93]. Thirdly, this study primarily focused on examining the effects of parent–child relationships on NCSs development. We also acknowledge that in real-life situations, the development of NCSs extends beyond the variables addressed in this study. Researchers can explore other factors such as the school and community environments, which may contribute to the development of adolescents’ non-cognitive skills. Finally, it is important to clarify that there is no common consensus on the measurement of NCSs. This study focused only on several positive aspects of NCSs. Future studies could expand the scope of NCSs components by including negative aspects as well.

## 8. Conclusions

It is noteworthy the closeness of the parent–child relationship with both parents decreased for secondary school students compared with primary school students and was accompanied by a decrease in grit, innovation, and conscientiousness levels and an increase in the number of positive friends. Male participants had closer relationships with their father and had a higher level of innovation, sense of competence and number of negative friends. Females were more socially anxious than males. These results highlight the importance of parents and teachers in closely monitoring the emotional needs and personality development of adolescents, especially those in secondary school and specialized education, based on gender differences.

Secondly, among Chinese adolescents, the levels of closeness in parent–child relationships and the number of positive friends show positive predictive relationships with grit and conscientiousness. These findings highlight the important role of close parent–child relationships and good positive friends in fostering later grit, innovation, conscientiousness, and hope. Furthermore, social anxiety negatively predicts innovation, conscientiousness, and hope, and the number of negative friends shows negative predictive relationships with grit and conscientiousness.

Thirdly, this study simultaneously examined the mediating roles of social anxiety and number of friends in both parental–child relationships and adolescent NCSs within the same model by controlling for grades and gender. The findings of the present study extend our insight into the mechanisms underlying the associations among the parent–child relationships, social anxiety, number of friends and NCSs of adolescents, supplementing previous relevant studies. Both mothers and fathers play a crucial role in the NCSs development of children, and the effect of a close relationship with fathers was even larger on conscientiousness, innovation and grit than with mothers. Both parents should take responsibility for their child’s education. It is crucial for both parents to support and cooperate with each other to establish a warm and harmonious family atmosphere and cultivate high-quality parent–child relationships. These endeavors not only contribute to increasing the number of positive friendships at school but also alleviate youth social anxiety and diminish the prevalence of negative peer relationships, which in turn promotes the development of children’s NCSs.

## Figures and Tables

**Table 1:** Descriptive statistics of parent–child relationships, non-cognitive skills, social anxiety and number of friends.

	1	2	3	4	5	6	7	8	9
1 FCR	1								
2 MCR	0.66 **	1							
3 Grit	0.17 **	0.17 **	1						
4 INO	0.20 **	0.16 **	0.52 **	1					
5 COS	0.26 **	0.27 **	0.65 **	0.60 **	1				
6 Hope	0.28 **	0.25 **	0.39 **	0.50 **	0.45 **	1			
7 SA	−0.30 **	−0.21 **	−0.07 *	−0.18 **	−0.13 **	−0.24 **	1		
8 NPF	0.11 **	0.11 **	0.19 **	0.14 **	0.18 **	0.26 **	−0.10 **	1	
9 NNF	−0.06	−0.15 **	−0.10 **	0.08 *	−0.09 *	−0.01	0.04	0.00	1
Total Mean	3.64 ± 0.92	3.75 ± 0.79	3.27 ± 0.61	3.11 ± 0.60	3.10 ± 0.56	4.20 ± 1.19	0.76 ± 0.47	2.66 ± 0.43	1.54 ± 0.53
Female	3.55 ± 0.88	3.72 ± 0.78	3.27 ± 0.58	3.03 ± 0.54	3.12 ± 0.50	4.11 ± 1.11	0.86 ± 0.45	2.69 ± 0.39	1.35 ± 0.42
Male	3.73 ± 0.95	3.78 ± 0.80	3.27 ± 0.65	3.18 ± 0.64	3.08 ± 0.61	4.30 ± 1.26	0.66 ± 0.46	2.64 ± 0.47	1.74 ± 0.56
Primary school	3.85 ± 0.91	3.90 ± 0.73	3.34 ± 0.54	3.23 ± 0.56	3.21 ± 0.50	4.26 ± 1.12	0.74 ± 0.48	2.60 ± 0.43	1.58 ± 0.48
Secondary school	3.52 ± 0.91	3.67 ± 0.81	3.23 ± 0.65	3.04 ± 0.61	3.04 ± 0.58	4.17 ± 1.23	0.78 ± 0.46	2.70 ± 0.43	1.52 ± 0.55
Min	1	1	1	1	1	1	0	1	1
Max	5	5	4	4	4	6	2	3	3
Alpha	0.89	0.85	0.76	0.69	0.83	0.89	0.87	0.77	0.78

Note. * *p* < 0.05 (2-tailed); ** *p* < 0.01 (2-tailed); FCR: Father–child relationship; MCR: Mother–child relationship; INO: Innovation; COS: Conscientiousness; SA: social anxiety; NPF: number of positive friend; NNF: number of negative friend.

**Table 2:** Standardized coefficients of SEM.

	Model 1 (SE)				Model 2 [95%CI]			
	Grit	INO	COS	Hope	Grit	INO	COS	Hope
Gender	−0.026 (0.026)	0.143 *** (0.025)	−0.078 ** (0.024)	0.091 *** (0.023)	0.029 [−0.028–0.085]	0.087 ** [0.028–0.141]	−0.028 [−0.081–0.024]	0.044 [−0.005–0.093]
Grade	0.129 *** (0.025)	0.164 *** (0.024)	0.197 *** (0.023)	0.092 *** (0.023)	0.139 *** [0.087–0.189]	0.150 *** [0.099–0.200]	0.188 *** [0.142–0.234]	0.055 * [0.009–0.100]
FCR	0.099 ** (0.032)	0.142 *** (0.033)	0.182 *** (0.03)	0.158 *** (0.029)	0.101 ** [0.027–0.175]	0.144 *** [0.068–0.223]	0.184 *** [0.114–0.255]	0.160 *** [0.088–0.235]
MCR	0.101 ** (0.031)	0.070 * (0.031)	0.128 *** (0.028)	0.183 *** (0.028)	0.102 ** [0.030–0.173]	0.071 [−0.004–0.147]	0.130 *** [0.064–0.195]	0.186 *** [0.116–0.256]
SA	−0.024 (0.027)	−0.134 *** (0.027)	−0.053 * (0.025)	−0.204 *** (0.023)	−0.025 [−0.087–0.038]	−0.137 *** [−0.199–0.074]	−0.054 [−0.113–0.004]	−0.209 *** [−0.263–0.154]
NPF	0.287 *** (0.029)	0.224 *** (0.03)	0.275 *** (0.028)	0.222 *** (0.027)	0.289 *** [0.225–0.353]	0.226 *** [0.155–0.295])	0.277 *** [0.214–0.339]	0.224 *** [0.163–0.284]
NNF	−0.137 *** (0.029)	0.054 (0.029)	−0.155 *** (0.027)	−0.005 (0.025)	−0.150 *** [−0.220–0.082]	0.059 [−0.012–0.131]	−0.169 *** [−0.234–0.104]	−0.006 [−0.067–0.055]
FCR-SA-NCSs	-				-	0.026 [0.013–0.043]	0.010 [−0.001–0.023]	0.040 [0.024–0.060]
MCR-SA-NCSs	-				-	0.012 [0.002–0.025]	0.005 [0.000–0.013]	0.019 [0.004–0.035]
FCR-NPF-NCSs	-				0.046 [0.022–0.070]	0.036 [0.017–0.056]	0.044 [0.022–0.067]	0.035 [0.017–0.056]
MCR-NPF-NCSs	-				0.024 [0.001–0.048]	0.019 [0.001–0.038]	0.023 [0.001–0.046]	0.019 [0.001–0.038]
FCR-NNF-NCSs	-				0.016 [0.005–0.029]	-	0.018 [0.005–0.032]	-
MCR-NNF-NCSs	-				0.022 [0.009–0.038]	-	0.025 [0.012–0.041]	-

Note. * *p* < 0.05 (2-tailed); ** *p* < 0.01 (2-tailed); *** *p* < 0.001 (2-tailed).

## Data Availability

The data is unavailable due to privacy or ethical restrictions.
